# Application of image recognition-based tracker-less augmented reality navigation system in a series of sawbone trials

**DOI:** 10.1186/s42836-024-00263-1

**Published:** 2024-08-02

**Authors:** Elvis Chun-Sing Chui, Kyle Ka-Kwan Mak, Randy Hin-Ting Ng, Ericsson Chun-Hai Fung, Harold Hei-Ka Mak, Mei-Shuen Chan, Wei Zhao, Xiuyun Su, Jin Zhang, Jianglong Xu, Hongxun Sang, Guoxian Pei, Michael Tim-Yun Ong, Wing-Hoi Cheung, Sheung-Wai Law, Ronald Man Yeung Wong, Patrick Shu-Hang Yung

**Affiliations:** 1 Department of Orthopaedics and Traumatology, The Chinese University of Hong Kong, Shatin, Hong Kong SAR, China; 2 Department of Orthopaedics, Southern University of Science and Technology Hospital, Shenzhen, 518055 China; 3 Department of Orthopaedics, Shenzhen Hospital of Southern Medical University, Shenzhen, 510086 China; 4 Department of Orthopaedics, Shenzhen Children’s Hospital, Shenzhen, 518026 China; 5 Department of Orthopaedics and Traumatology, Prince of Wales Hospital, Shatin, Hong Kong SAR, China

**Keywords:** Augmented reality, Computer-assisted navigation system, Computer-assisted surgery, Mixed reality, Surgical navigation

## Abstract

**Background:**

This study introduced an Augmented Reality (AR) navigation system to address limitations in conventional high tibial osteotomy (HTO). The objective was to enhance precision and efficiency in HTO procedures, overcoming challenges such as inconsistent postoperative alignment and potential neurovascular damage.

**Methods:**

The AR-MR (Mixed Reality) navigation system, comprising HoloLens, Unity Engine, and Vuforia software, was employed for pre-clinical trials using tibial sawbone models. CT images generated 3D anatomical models, projected via HoloLens, allowing surgeons to interact through intuitive hand gestures. The critical procedure of target tracking, essential for aligning virtual and real objects, was facilitated by Vuforia’s feature detection algorithm.

**Results:**

In trials, the AR-MR system demonstrated significant reductions in both preoperative planning and intraoperative times compared to conventional navigation and metal 3D-printed surgical guides. The AR system, while exhibiting lower accuracy, exhibited efficiency, making it a promising option for HTO procedures. The preoperative planning time for the AR system was notably shorter (4 min) compared to conventional navigation (30.5 min) and metal guides (75.5 min). Intraoperative time for AR lasted 8.5 min, considerably faster than that of conventional navigation (31.5 min) and metal guides (10.5 min).

**Conclusions:**

The AR navigation system presents a transformative approach to HTO, offering a trade-off between accuracy and efficiency. Ongoing improvements, such as the incorporation of two-stage registration and pointing devices, could further enhance precision. While the system may be less accurate, its efficiency renders it a potential breakthrough in orthopedic surgery, particularly for reducing unnecessary harm and streamlining surgical procedures.

## Introduction

Conventionally, HTO was carried out without the involvement of computer-assisted navigation system (CAS), which had been associated with inconsistency of the postoperative alignment of the mechanical axis as it does not achieve accurate opening or closing of the wedges, preoperative planning with high precision or desired degree of control over intraoperative re-alignment. It had also been associated with other issues, including the inaccurate positioning of the hinge axis and dis-orientation of surgical instruments (e.g., chisel and electric saw), which resulted in unnecessary damages to the neurovascular structures and tibia plateau [1–3]. To overcome the numerous shortcomings of the conventional HTO approach, the involvement of computer-aided navigation system was warranted as it was commonly used in surgical operation in which the system provided information such as the spatial location and orientation of the medical instrument and pre-planned virtual path would be displayed via computer screens [4]. One of the examples of computer-aided navigation system was Stryker navigation system which had been widely utilized in the surgical setting.

According to the article, they utilized the VectorVision system—one of the CAS technologies. When the system was applied in close-wedge HTO, it was able to provide vital information including the level of osteotomy, angle of correction, size of the wedge and deformity of the tibia. The system also consisted of a pre-planned drill guide which was utilized by surgeons to position K-wires into proximal and distal planes of the osteotomy, followed by the osteotomies over the K-wires in the two corresponding planes and the attachment of the two sites of osteotomy by fixatives such as Miniplate Staple. The postoperative mechanical axis alignment displayed on the computer screen was confirmed with measurements obtained from radiographs [1]. However, surgeons will have to switch between the computer screen and the operative sites during the operation, which contributed to inefficiency and inconveniences [4]. These shortcomings led to the emergence and development of AR navigation system as it allowed for the combination of real-world and virtual information such as auditory and visual stimuli and such phenomenon could be visualized via head-mounted display (HMD) device such as Microsoft HoloLens which were worn by surgeons. In recent years, AR navigation system has been employed in a wide range of medical specialities such as brain, spine and orthopaedic surgeries and desirable clinical outcomes have been observed [5, 6].

When the system was applied in orthopaedic surgeries such as lateral temporal bone resection (LTBR), the surgeon was able to perform the sophisticated surgical procedures at ease without any intraoperative complications since they were able to (1) visualize the anatomy of the interior and exterior of the relevant structures, (2) visualize the models of all surgical steps simply by hand gestures and (3) gain more experience from all the preoperative practices of LTBR with the aid of the AR navigation system [7]. Despite these, it lacked the ability of overlaying technique and three-point registration which enabled the complete alignment between the operative region and the 3D holograms (displayed on HMD)—imperative for overall surgical performance, including accurate positioning of surgical instruments to avoid damages to vital internal structures intraoperatively. Therefore, the incorporation of MR is essential for desirable clinical outcomes of surgeries, with certain extent of complexity such as right thoracoscopic lingular wedge resection where the effect of lung deflation could be mimicked due to the simulation enabled by MR, which allowed surgeons to accurately identify the location of the metastatic tumours [8]. This was possible as MR allowed for (1) the overlaying between the real object and virtual object and (2) connection between them so that they could interact with each other [9].

In the previous work on comparison in performance of Stryker navigation system (conventional navigation system) and personalized surgical guides in HTO bone model trial, inefficiencies and inaccuracies of conventional navigation system were observed and could be addressed by AR-MR navigation system given its numerous advantages in the surgical setting. In this study, a novel AR-MR navigation system was developed and assessed in three aspects (preoperative planning time, intraoperative time and accuracy) in pre-clinical bone model trials where HTO was conducted.

## Materials & methodology

### AR navigation system

The navigation system was comprised of HoloLens 2 (Microsoft, Redmond, WA, USA), Unity Engine (Unity Technologies, San Francisco, CA, USA) and Vuforia software (PTC, Boston, MA, USA). Aim-position tracking device was capable of marking points coordinates with high precision and of tracking 200 markers simultaneously. HoloLens 2 served as the head-mounted display (HMD) and consisted of a see-through holographic lens, multiple visible light and infra-red cameras for environment sensing, thus enabling the applications of AR and Virtual Reality (VR). Unity Engine was chosen as the platform for the development of application which would be displayed on Microsoft HoloLens 2. Vuforia was used to develop the object tracking functionality as it was the only viable development tool and runtime environment for the Unity and HoloLens [10].

### Operating interface (Microsoft HoloLens)

CT images of the patients were acquired to generate the 3D anatomy of the high tibial bone. The 3D anatomical models and user interfaces were projected virtually through the HMD. The models could be visualized from different angles. Hand tracking was the main user input to the HoloLens. The users could interact with the HoloLens application through a set of hand gestures.

### Target tracking

**Fig. 1:**
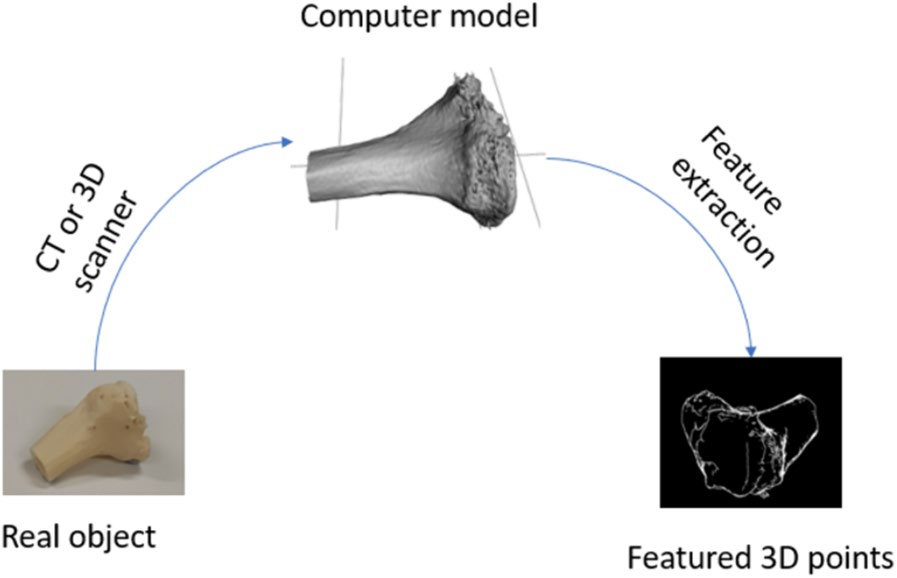
Description of target tracking where the real object was first converted to computer model through CT or 3D scanner and subsequently into featured 3D points through feature extraction

The ability of target tracking was rendered by the feature detection algorithm of Vuforia software (shown in Fig. 1). The feature detection formula was as follows: set the error of the ith pair of points is ${e}_{i}$, then ${e}_{i}={p}_{i}-(R{p}_{i}^{\prime}+t)$, where ${p}_{a}$ is a featured point of the holograph, ${p}_{i}$ is a point of the real object, *R* is the rotation matrix and *t* is the transfer vector (${\text{x}}_{1}$, ${\text{y}}_{1}$, ${\text{z}}_{1}$).

**Fig. 2:**
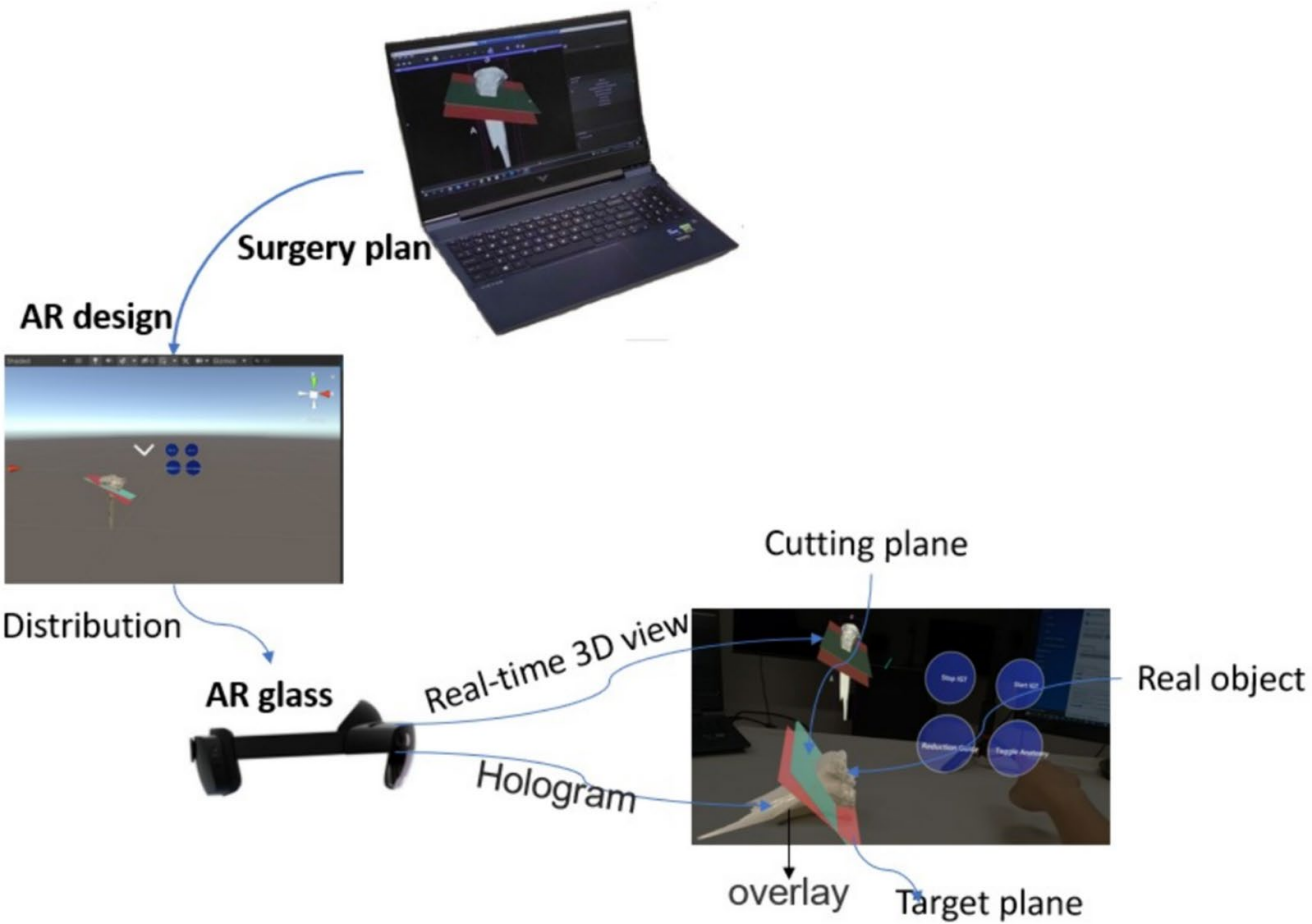
The overlaying between real-world and virtual bones enabled surgeons to achieve the identification of the incision site (indicated by green plane), real-time visualization of the HTO operation and provision of spatial information to guide surgeons to align the cross-sectional region of the proximal tibial bone with the target plane (indicated by red plane) perfectly, resulting in desirable mechanical axis alignment

At first, the three-dimensional (3D) digital representation of the “real object” was acquired with the use of computed tomography (CT) or 3D scanner. Then, as the 3D representation was processed, it led to the production of computer model whose contour was used to extract the featured 3D points. At last, the feature detection algorithm capable of connecting the featured 3D points and their corresponding points of the real-world object enabled the “overlaying phenomenon”, which was the complete alignment between the holograph and real-world object. The overlaying formula was as follows: ${\text{min}_{R,t}}J=\frac{1}{2}{\sum }_{i=1}^{n}{|{p}_{i}-(R{p}_{i}^{\prime}+t)|}^{2}$. When overlaying between the real-world and virtual bones was achieved, Microsoft HoloLens enabled (1) the surgeons to locate the incision site (indicated by cutting planes mounted on the proximal tibial region in virtual setting), (2) the visualization of HTO operation in real-time as incisions/cuts were being made along the cutting planes and (3) the provision of spatial information to achieve the desired mechanical axis alignment (shown in Fig. 2).

### Clinical assessment of the AR navigation system

To investigate the capability of the navigation system in carrying out target tracking and overlaying techniques, 30 tibial sawbone models were generated by 3D printing according to CT images of 10 HTO patients. The sawbones were then used by an orthopaedic surgeon to perform high tibial osteotomies in three different experimental conditions: HTO with metal personalized surgical instrument (“HTO-mPSI”), HTO aided with Stryker Navigation system (“conventional navigation system”) and HTO aided with the AR-based navigation system (“HTO-AR”) where 10 sawbones were used in each condition. In addition, 10 metal PSI were designed according to CT images of 10 HTO patients and subsequently generated by 3D printing.

Throughout the trials, three parameters were examined: preoperative planning time, intraoperative time and accuracy in all conditions. Preoperative planning time was the amount of time needed either to design and manufacture the surgical guides/personalized surgical instruments or to design surgical tools (cutting planes and screws) and place them in the right geometrical location of the tibial bone in the DICOM image. Intraoperative time was the amount of time needed to perform the whole surgical procedure, including the placement of tools (e.g., surgical guides, pins) and target tracking of both navigation systems. Accuracy was examined by the difference in angles between the preoperatively planned cutting planes and the planes on which the actual cuts were made along intraoperatively in 2 axes (XZ- and YZ-axis).

### Workflow of surgical guides (metal)-assisted HTO

During the preoperative planning phase, the manufacturing procedure of the surgical guides was as follows: At first, a 3D scanner (Shining Einscan Pro 2x) was used to scan the sawbones and the result was output as an STL file. The STL file was then imported into 3-Matic Software (Materalise, Belgium) where designing process of surgical guides was carried out. The surgical guides were customized according to the unique characteristics of tibial bones of different patients. At last, the surgical guides were manufactured by the metal 3D printing technique.

**Fig. 3:**
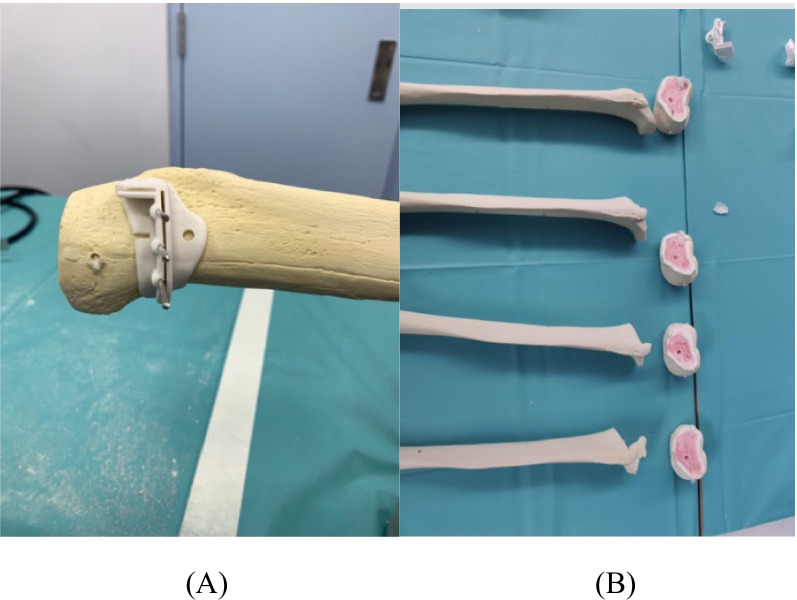
Illustration of surgical guides. **A** Main cut was made along the vertical tunnel of surgical guide whereas side cut was made along the horizontal tunnel of surgical guide (**B**) After two cuts were made, the proximal tibial regions were removed

To start with the high tibial osteotomy, screws with a diameter of 2 mm were placed into 3 holes of surgical guide. It was followed by the two cuts (main cut and side cut) made along the XY-axis to the extent that the proximal tibial region was removed. Main cut was made along vertical tunnel of surgical guide whereas the side cut was made along horizontal tunnel of surgical guide (shown in Fig. 3).

### Workflow of conventional navigation-assisted HTO

Initially, the sawbones were scanned by Ein-scan 3D hand-held scan (Shining 3D, Hangzhou, China) and subsequently underwent segmentation via Mimics software (Materalize, Leuven, Belgium). It was ensued by the designing of 2 cutting planes and 6 screws, which would be introduced into the proximal tibial region in DICOM files. At last, the DICOM files consisting of the tibial bones mounted with cutting planes and screws were inputted into the “OrthoMap Module” of the conventional navigation system (Stryker eNlite Navigation system).

**Fig. 4:**
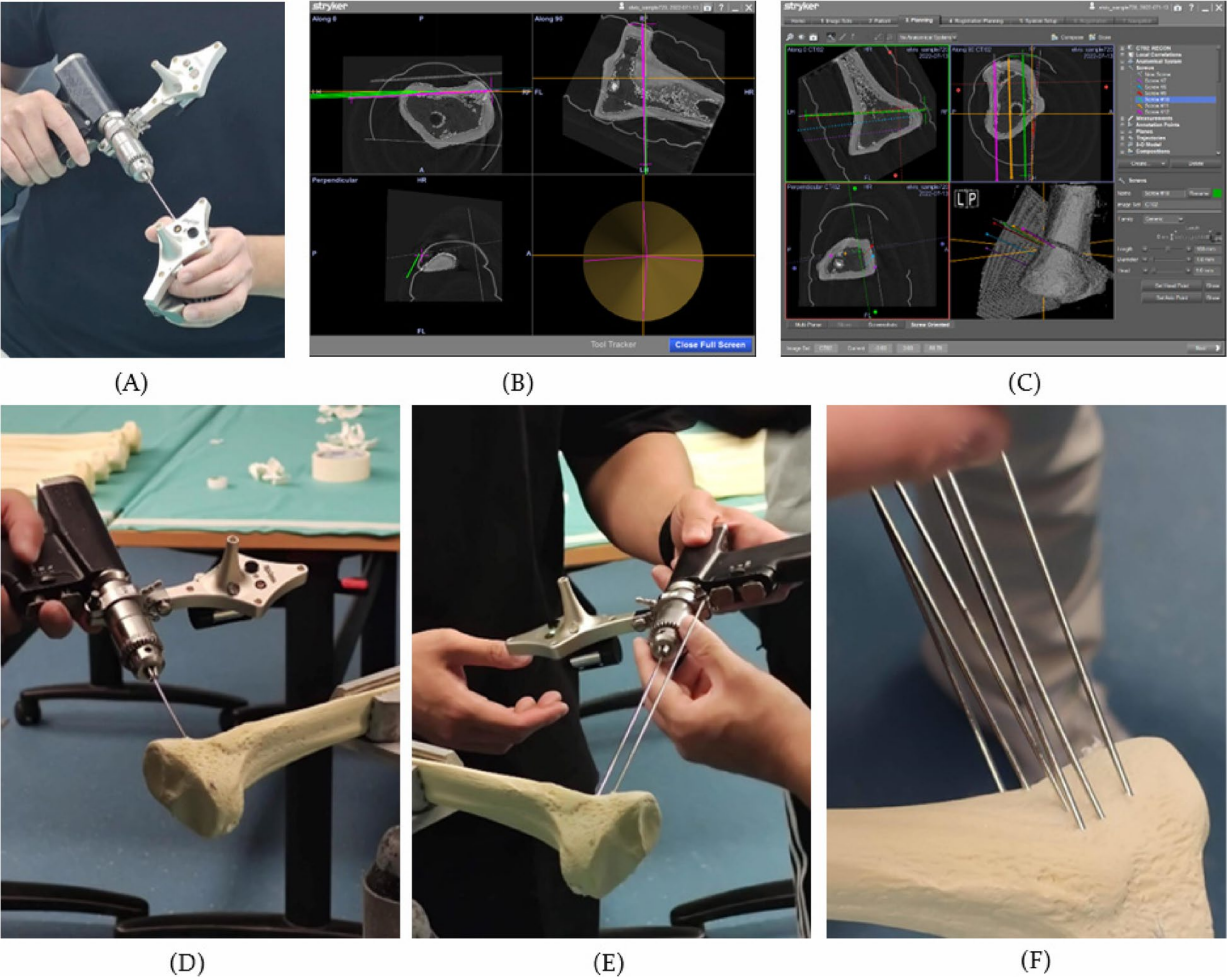
Demonstration of conventional navigation-assisted HTO

During the intraoperative phase, five-point matching and surface matching were conducted in the “OrthoMap Module” of the conventional navigation system. This enabled the addition of six screws in topographic location (with respect to the two incision sites of surgical guide; 3 screws along each incision site) of the bones and two cuts (main cut and side cut) were then made along these screws. The steps of conventional navigation-assisted HTO are illustrated in Fig. 4. At the beginning, the calibration and matching procedure of the navigation system were performed. With the aid of the navigation system on the screen, the screws/pins were inserted onto the proximal tibia region to form the site of main cut and side cut.

### Workflow of the AR navigation-assisted HTO

**Fig. 5:**
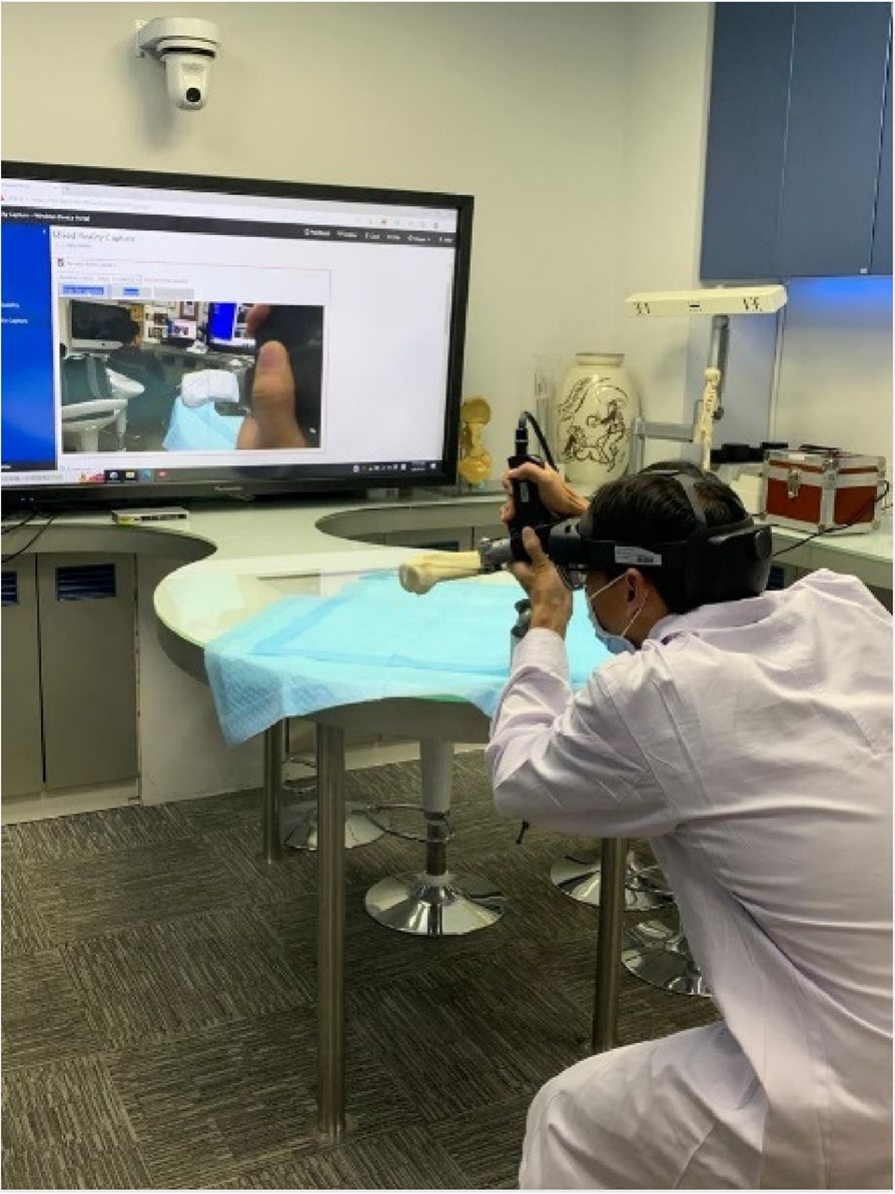
Illustration of target tracking. When the tibial bone was within the visual field of Microsoft HoloLens (worn by the orthopaedic expert), target tracking was enabled to achieve the overlaying

At first, the tibial bones were placed within the visual field of AimPosition tracking device. It was followed by uploading 3D “modified” holograms into HoloLens 2. Target tracking was carried out and resulted in overlaying of the virtual and real tibial bones within the Microsoft HoloLens where the surgeon was able to visualize the virtual path through which the electric saw should move along to make incision (shown in Fig. 5).

## Results

**Fig. 6:**
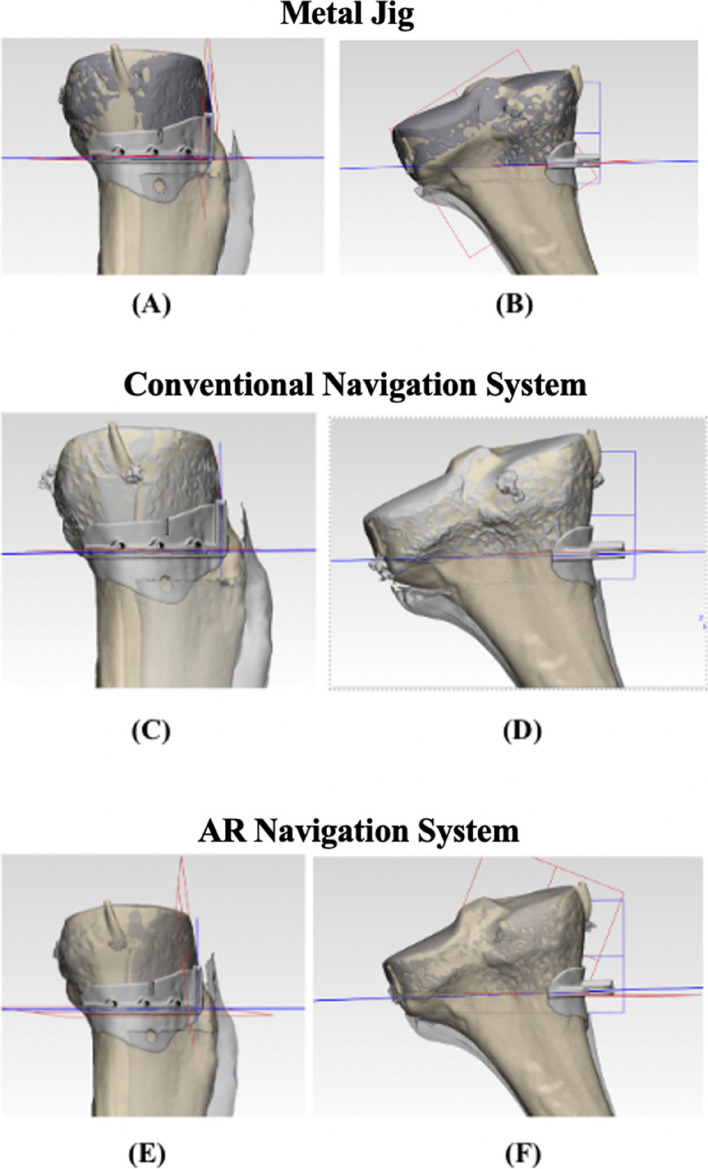
Illustration of angle differences in YZ- and XZ- planes. Blue plane shows the surgical planned cutting plane while red plane is the practical model cutting plane. **A**, **B** demonstrate the angle difference between red and blue cutting planes when a metal jig was used. **C**, **D** illustrate the angle difference between red and blue cutting planes when conventional navigation system was used. **E**, **F** exhibit the angle difference between red and blue cutting planes when AR navigation system was employed

**Fig. 7:**
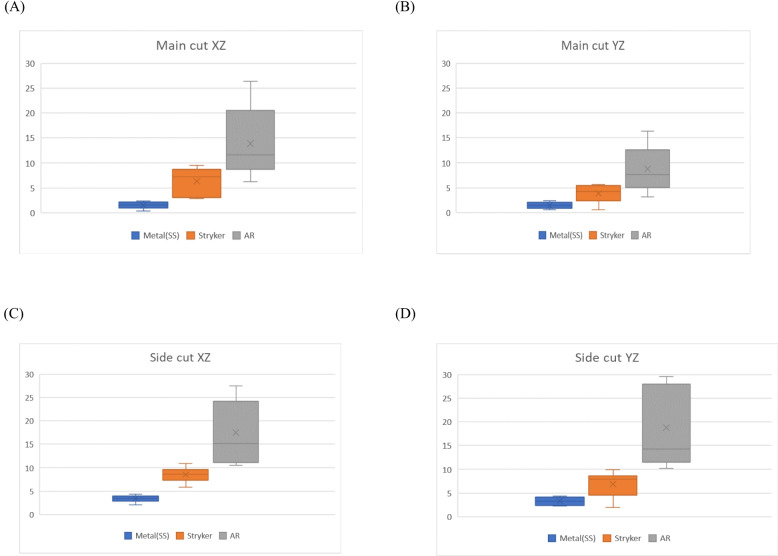
Demonstration of angle differences of metal jig, conventional navigation system and AR navigation system. X-axis displays the three items under investigation while y-axis represents the angle difference (i.e., the lower the value, the higher the accuracy of incision). **A**, **B** display the angle differences during the main cut (vertical cut) while (**C**, **D**) shows the angle differences during the side cut (horizontal cut)

With AR navigation system, it took only 4 min to complete preoperative planning. However, the preoperative time of planning for conventional navigation system and surgical guides were 30.5 min and 75.5 min, respectively. With regard to the intraoperative time, the AR navigation system only took 8.5 min, on average, to finish the bone cutting. In comparison, the intraoperative time for conventional navigation system and surgical guides were 31.5 min and 10.5 min, respectively. Lastly, in terms of the accuracy, AR navigation system < conventional navigation system < metal 3D printing. The data collected for each parameter except accuracy are displayed in Table 1. The angle differences between the preoperatively planned cutting plane and actual intraoperative cutting plane in the three different circumstances are illustrated in Fig. 6. To better present the difference in angles, a figure containing box and whisker plots was drawn (Fig. 7).

**Table 1:** Comparison of AR navigation system, conventional navigation system and metal 3D printing

	**AR navigation system**	**Conventional navigation system**	**Metal (3D printing)**
Preoperative Planning time (average) (mins)	4	30.5	75.5
Manufacturing time (business days)	N/A	N/A	7
Delivery time (business days)	N/A	N/A	12
Material cost (USD) per kilogram	N/A	N/A	150
Intraoperative time (mins)	8.5	31.5	10.5
Average angle differences (degrees)	14.7	6.41	2.40

*N/A* not applicable

## Discussion

### Preoperative time

Our findings revealed that the preoperative planning time and intraoperative time were minimized when the AR navigating system was utilized to carry out HTO in comparison with two other modalities. To start with, AR navigation-assisted HTO was relatively easier to set up and the operating interface was easy to manoeuvre. Utilizing the hand gesture interaction, the user could control the system immediately and conveniently. Moreover, the target tracking system was developed by using the Vuforia software and fully capable of tracking the target bone efficiently. The virtual cutting planes which overlaid the actual tibial bone were automatically generated, which further shortened the preoperative planning time. In contrast, conventional navigation-assisted HTO involved numerous preparatory steps prior to the commencement of the operation. It included scanning of tibial sawbones, mimics segmentation, designing of the cutting planes and positioning of screws. These procedures were relatively time-consuming, which led to a longer preoperative time. Furthermore, HTO-mPSI had the longest preoperative time among the three modalities since it took time for the jigs to be designed, manufactured and delivered.

### Intraoperative time

With regard to the intraoperative time, HTO-AR only involved cutting the bones according to the virtual cutting plane, which made the manual work during operation fast and simple. On contrary, HTO-mPSI involved not only the manual cutting but also the placement of surgical guides and pins, which led to prolonged intraoperative time. However, the conventional navigation system was more sophisticated since steps such as point-matching and calibration, which were time-consuming. More work has to done to enhance the accuracy of the overlaying procedure. In addition, insertion of pins was also required prior to the osteotomy. Therefore, use of the conventional navigation system took the longest time.

### Accuracy

In terms of accuracy, AR-assisted system was the least accurate, which could be explained by the fact that AR technology is still on its early stage. Since application of AR in clinical practice was a recent development, the technology, in some ways, was immature, which affects the stability of the system. For instance, the AR navigation system, sometimes, failed to track the orientation and the position of the real bone model accurately. The virtual tibial bones were not able to perfectly align with the actual bones, resulting in inaccurate positioning of the cutting plane, which might be mainly culpable for the inaccurate bone incision. Moreover, the conventional navigation-assisted HTO involved the procedure of screw insertion before actual bone cutting whereas AR-assisted HTO required direct bone cutting without the aid of any surgical guide or pin. In addition, surgical guide generated by Metal 3D printing technique was most accurate compared to the navigation-assisted systems. Since the insertion of a pin into the bone model was guided by a Metal jig, the positional error was lessened. Hence, the error associated with each incision was kept at minimal since the metal surgical guides were rigid and had a high melting point [11, 12]. Given its inherent physical properties, structural deformity or collapse was unlikely during the cutting process. Therefore, Metal 3D-printing surgical guides-assisted HTO was the most accurate amongst all surgical guidance tools.

### Alternative AR navigation systems

Similarly, other AR navigation systems were available and had the capability of target tracking in surgical setting. Currently, AR navigation systems have been widely used as a means to facilitate complicated and sophisticated surgical procedures. Previous studies showed that AR navigation system visual display could be achieved variously and and the target tracking functionality could be built differently so that the system could be tailored to different purposes. Liu et al. reported an AR navigation system built for telementoring (i.e., remote medical training), which could address the problem of relative unavailability of medical experts in remote areas. The AR navigation system used Microsoft HoloLens for display and target tracking was achieved by accurately positioning two metal–oxide–semiconductor (CMOS) cameras around the operative field, developing a tracking coordinate system and fine-tuning the extrinsic matrices of the CMOS camera by placing four fiducial markers at specific locations that were in proximity to the four corners of the operation table [13]. In another instance, the AR navigation system was employed in animal studies and was composed of an IR reference marker fixer, a sensing camera and a miniature pico-projector (54 mm × 170 mm × 21 mm). The projector was utilized to display 3D images. The real-time target tracking was enabled via an optical sensor mounted on the surface of the pic-projector and the ensuing calibration procedures that could definitively identify the spatial relationship between the optical sensor and the central point of the projector [14].

Despite the promising benefits resulting from the incorporation of target tracking in other existing AR navigation systems, it is imperative to ensure that the system possesses high precision to minimize unnecessary harm to the patients while maintaining efficiency, as illustrated by the following studies. Peng et al. utilized an augmented reality (AR) system comprising a Microsoft HoloLens and a scalpel equipped with a 3D point tracking device. This setup enabled tele-mentoring, where the movements of the scalpel used by an experienced surgeon were transmitted and visualized in real-time on the HoloLens worn by an inexperienced surgeon who was performing operation in another (usually a remote) site. This allowed the inexperienced surgeon to perform surgeries under virtual guidance, with minimal guidance errors (< 2.75 mm) and tracking errors (< 2.5 mm). Its practical use was validated through two forms: phantom validation (use of arm model) and in-vivo validation where skin grafting and fasciotomy were performed on a rabbit model with desirable pre-clinical outcomes. This will greatly enhance surgical quality whilst avoiding unnecessary hazards to patients in remote areas [13]. In another study, Gongseng and the team developed an AR-assisted radiotherapy positioning system to deal with the difficulties associated with positioning of the patients for radiotherapy with the conventional approaches (cone beam computed tomography [CBCT] and MRI-Linac), including the impossibility to give positioning guidance in real-time, human errors-related fatigues and additional radiation dosage. Their system was capable of isocentre calibration, establishment of coordinate systems between the real world and virtual targets and object tracking. When the system was subjected to the anthropomorphic phantom test, the positioning errors were around 3.1 mm, 3 mm and 4.6 mm along the X, Y and Z axes respectively. Despite the fact that the outcome has yet to meet the clinical requirements but is promising if further enhancements, such as the incorporation of artificial intelligence models is enacted. The approach of this AR navigation system was advantageous in terms of reduced financial cost due to lower frequency of CBCT and improvement in healthcare quality resulting from a continuous spatial monitoring of the patients for correct positioning, which is not possible with CBCT [15].

### Enhancement of system performance

Some elements can be introduced to enhance the performance of the AR navigation system other than targeting tracking capability. One example is the two-stage registration procedure, point matching and surface matching [16], the former allowing for overlaying between virtual and real objects at lower quality and the latter further enhancing the overlaying phenomenon. Point matching is the placement of three to seven points upon surface of a real object and subsequently the corresponding points on virtual object will be mathematically determined by singular value decomposition (SVD) algorithm, which produces a coarse transformation matrix between the real and virtual objects [17]. The initial transformation matrix will undergo further refinement through surface matching. This process involves randomly placing hundreds of points on the physical object and determining the corresponding points on the virtual model using the iterative closest point (ICP) algorithm. As a result, the registration accuracy will be significantly improved by finalizing the transformation matrix [18]. Incorporating two-stage calibration is crucial for improving system performance as overlaying phenomenon rendered by target tracking will not be better than that rendered by two-stage registration.

Lastly, the use of pointing device (PD), which is comprised of three parts—notch, handle and tip, by which fiducial marker can be placed on the notch for geometrical localization and the tip is used for point registration and surface registration [19]. It also reduces the complexity of the operating system as the tracking device (in this case, AimPosition) will be replaced by the pointing device so AimPosition tracking device will not be required for the registration.

The benefits of the two-stage registration were demonstrated by a study where active (Stryker navigation system) and passive (VectorVision Sky, BrainLAB) neuro-navigation systems were compared in terms of four accuracy parameters (software accuracy, imaging accuracy, system accuracy and navigation accuracy). Both commercial navigation systems underwent two-phase registration procedure by following a strict and thorough experimental protocol on an anthropomorphic head phantom that physically mimics human skin. Amongst the four accuracy parameters, navigation accuracy was the ultimate parameter that represented the performance of each system as it took all of the possible errors into account. It was measured by mean Euclidean deviation and it was found to be 1.45 ± 0.63 mm for Stryker and of 1.27 ± 0.53 mm for BrainLAB respectively, which was relatively smaller than that of the above-mentioned AR navigation systems (Peng et al. and Gongseng et al.) without two-stage registration properties/features [13, 15, 20]. This also suggests that AR navigation system might have potential to be applied in orthopaedic surgeries considering that the overlaying can be accurately carried out by incorporating the two-stage registration procedure.

### Limitations of the AR navigation system

While performing the operation, WIFI connection was needed for the basic functioning of the HoloLens, the connection of WIFI may affect the overlaying phenomenon. Other external factors such as the brightness of the room, the surface of the operating table and surrounding soft tissue of the bone may interrupt the signal transmission of AR navigation system. Apart from that, dexterity of the cutting skills possessed by the surgeons was one of the requirements to achieve desirable results as no K-wire or jig or any assisting tools were involved in the bone model trials. It may be difficult for the inexperienced surgeons to visualize the most appropriate path where the osteotomy is performed.

### Limitations of the current study

This study had some limitations. Firstly, the samples that determine whether the outcome was true finding or not were not adequate. Due to high variability of the dataset, there was an increase in the margin of error and subsequently a decrease in the statistical power which contributed to skewed outcomes [21]. Besides, the Inter-operator variability—there were three orthopaedic specialists involved in the bone model trial—could significantly influence the accuracy and reliability of the results due to the differences in ways that osteotomies were performed and in their clinical experience.

### Further investigation

In future, further investigations can go in two directions: (1) application of the AR navigation system incorporated with pre-trained deep learning models and the comparison between two-stage registration procedure and the existing navigation system in pre-clinical trials in which anthropomorphic phantom with human-like internal organs, complex vasculatures and tissues will be used as in-vivo simulations of high tibial osteotomy; (2) Other than high tibial osteotomy, it can be applied in other medical fields such as neurosurgery which requires precise guidance procedures provided by the AR navigation system.

## Conclusion

Our findings showed that AR navigation system required the shortest preoperative planning time and intraoperative time, which significantly shortened the HTO surgical time. In addition, without the need of screw insertion, the AR navigation system reduced unnecessary harm to the tibial plateau and neurovascular systems [1–3]. Despite the fact that AR navigation system was the least accurate, it presented an accuracy similar to the navigation system (Stryker) and its use in orthopaedic surgeries is possible when further modifications and refinements are made. To improve the accuracy of bone cutting, modifications of image recognition techniques along with the introduction of two-stage registration techniques and pre-trained AI model for object contour identification are needed to improve the stability and target tracking capability of the AR navigation system.

## Data Availability

The datasets used and/or analyzed during the current study are available from the corresponding author on reasonable request.

## Abbreviations

3D: Three-dimensional
AR: Augmented Reality
CAS: Computer-assisted navigation system
CT: Computed tomography
HMD: Head-mounted display
HTO: High tibial osteotomy
HTO-AR: HTO aided with the AR-based navigation system
HTO-mPSI: HTO with metal personalized surgical instrument
ICP: Iterative closest point
LTBR: Lateral temporal bone resection
MR: Mixed reality
PD: Pointing device
SVD: Singular value decomposition
VR: Virtual Reality
